# Effects of Food Types, Frying Frequency, and Frying Temperature on 3-Monochloropropane-1,2-diol Esters and Glycidyl Esters Content in Palm Oil during Frying

**DOI:** 10.3390/foods10102266

**Published:** 2021-09-24

**Authors:** Jinglin Zhang, Wendi Zhang, Yuanzheng Zhang, Mingquan Huang, Baoguo Sun

**Affiliations:** 1 College of Food Science and Engineering, Tianjin University of Science & Technology, Tianjin 300457, China; zhangjinglin@btbu.edu.cn; 2 Key Laboratory of Brewing Molecular Engineering of China Light Industry, Beijing Technology & Business University (BTBU), Beijing 100048, China; 2030302049@st.btbu.edu.cn (W.Z.); 2128003403@btbu.edu.cn (Y.Z.); sunbg@btbu.edu.cn (B.S.); 3 Beijing Key Laboratory of Flavor Chemistry, Beijing Technology & Business University (BTBU), Beijing 100048, China

**Keywords:** palm oil, kinetic, frying model, influence factors, 3-MCPDE, GE

## Abstract

3-Monochloropropanediol esters (3-MCPDE) and glycidyl esters (GE) have high toxicity and have drawn global attention because of their widespread occurrence in refined oils and oil-based foods. In this study, the effects of food type (potato chips and chicken breasts), frying frequency, and frying temperature on the formation of 3-MCPDE and GE in palm oil (PO) were investigated. The results showed that 3-MCPDE was formed easier in chicken breasts than potato chips. The GE content decreased in PO after it was used for frying potato chips and chicken breasts with or without NaCl. Frying frequency was an influencing factor in the formation of 3-MCPDE and the decrease in GE in PO. Frying temperature was positively correlated with GE degradation, while it had a bidirectional effect on the formation of 3-MCPDE. The formation kinetic equations indicated that 3-MCPDE and GE followed zero-order reactions in PO. The estimated activation energy (Ea) of 1,2-bis-palmitoyl-3-chloropropanediol (Pa-Pa, 41.05 kJ/mol) was lower than those of the other three types of 3-MCPDE; this is the first theoretical explanation for why PO contains more 3-MCPD than other edible oils. Among GEs, glycidyl oleate (Li-GE) was degraded more readily than other GEs.

## 1. Introduction

Elucidating the formation of 3-monochloro-1,2-propanediol esters (3-MCPDE) and glycidyl esters (GE) during heat processing of frying foods is vital for reducing risks to human health. Although the toxicology of 3-MCPDE and GE has rarely been investigated, the free forms of 3-MCPD and glycidol have been classified as group 2B (possible human carcinogens) and group 2A (probably carcinogenic to humans) carcinogens by the International Agency for Research on Cancer (IARC), respectively [1]. In addition, recent scientific studies have indicated that 3-MCPD and glycidyl are present in the human digestive tract [2]. In fact, a large part of the MCPD in foods is esterified with fatty acids, while a very small part is free. 3-MCPDE and GE have been reported in biscuits, infant formulas, and various types of vegetable oils, especially refined palm oil. The content range of 3-MCPDE was from ND to 10 mg/kg and that of GE was from ND to 9.1 mg/kg [3,4,5,6,7].

During the last decade, researchers have investigated the content of 3-MCPDE and GE in oils during the refining process and during food processing. 3-MCPDE and GE were significantly increased in deodorization, one of the refining steps [8]. In addition to being generated in refined oil, 3-MCPDE and GE in oil could migrate into foods during food processing. Owing to its rapid speed and simplicity, deep-frying has been widely used in food processing. Recent studies have found that fried foods contain higher concentrations of 3-MCPDE and GE than other types of foods. These contaminants migrate to fried foods from frying oil rather than being formed in the foods themselves during frying [9]. Therefore, it is worthwhile to study the changes in the content of 3-MCPDE and GE in oil during frying.

Many factors, such as heating temperature, heating time, and NaCl amount, play an important role in the formation of 3-MCPDE and GE during the deodorization stage of oil refining. 3-MCPDE and GE form easily under high temperature and low moisture conditions because the frying oil is decomposed into diacylglycerols (DAGs) and monoacylglycerols (MAGs) by hydrolysis, polymerization, or oxidation. DAGs and MAGs are the most important precursors of 3-MCPDE and GE. Chlorides are also precursors of 3-MCPDE and can promote the formation of GE [10,11]. Researchers have found that total polar components (TPC) and color value are highly correlated with 3-MCPDE content [12]. There are also some relationships between the degree of oxidation of the frying oil and the amount of 3-MCPDE and GE [13].

Apart from the above influencing factors, many other components present in food may contribute to the formation of 3-MCPDE and GE during heat treatment. For example, researchers found that food type has an important effect on the formation of 3-MCPDE and GE during the frying process. After frying chicken breasts for 100 min per day for five consecutive days, Wong et al. found that the content of 3-MCPDE in the frying oil was significantly increased while that of GE was decreased [13]. However, an opposite trend for the content of 3-MCPDE and GE occurred in fried potato chips [14]. When different raw materials (meat, cereals, fruit, bulbs, and tubers) were fried in palm oil containing 1.6 mg/kg of 3-MCPDE, the amount of 3-MCPDE in all of the fried products varied between 0.12 to 0.25 mg/kg [9]. In addition, different fatty acid compositions may influence the amount of 3-MCPDE or GE. Most of the unrefined oils contained undetectable concentrations of 3-MCPDE and GE, but the refined palm oil and palm olein samples had higher concentrations of both 3-MCPDE and GE than other types of oils. The average content of GE ranged from below the limit of quantification (LOQ) to 4.09 mg/L for glycidyl palmitate (Pa-GE) and below LOQ to 8.42 mg/L for glycidyl linoleate (Ol-GE). The average content of 3-MCPDE ranged from below LOQ to 1.92 mg/L for 1,2-bis-palmitoyl-3-chloropropanediol (Pa-Pa), 0.023 mg/L to 5.05 mg/L for palmitoyl-oleoyl-3-chloropropanediol (Pa-Ol), and 0.028 mg/L to 2.11 mg/L for 1,2-bis-oleoyl-3-chloropropanediol (Ol-Ol) in refined palm oil [15].

Although previous studies have evaluated the content changes of 3-MCPDE and GE in the frying process, the influences of various foods and frying frequency on 3-MCPDE and GE are not clear. Moreover, researchers have not fully studied the effects of different glycerin esters on the changes in 3-MCPDE and GE. Therefore, the present study investigated the effects of food type, frying frequency, and frying temperature on the formation or degradation of 3-MCPDE and GE during the frying process. The color and TPC were measured to establish their relationship with 3-MCPDE or GE. Kinetic models of 3-MCPDE and GE were also established to further understand their formation and degradation rules. Reaction kinetics are a powerful tool for expounding chemical mechanisms that have been applied to 5-hydroxymethylfurfural, acrylamide, β-carboline heterocyclic amines, and advanced glycation end products in food research [16,17,18]. The results of this study would help to control the levels of 3-MCPDE and GE during the frying process.

## 2. Materials and Methods

### 2.1. Chemicals

Isopropanol (IPA) and methanol (HPLC grade) were acquired from Merck KGaA (Darmstadt, Germany). Formic acid (HPLC grade) was purchased from Mreda Technology Inc. (Beijing, China). Ammonium formate (LC/MS grade) was obtained from Fisher Scientific (Waltham, MA, USA).

Deuterated internal standards, including glycidyl oleate-*d*_5_ (Ol-GE-*d*_5_), glycidyl stearate-*d*_5_ (St-GE-*d*_5_), 1,2-bis-palmitoyl-3-chloropropanediol-*d*_5_ (Pa-Pa-*d*_5_), and 1,2-bis-oleoyl-3-chloropropanediol-*d*_5_ (Ol-Ol-*d*_5_); 3-MCPDE standards, including 1,2-bis-palmitoyl-3-chloropropanediol (Pa-Pa), oleoyl-palmitoyl-3-chloropropanediol (Ol-Pa), 1,2-bis-oleoyl-3-chloropropanediol (Ol-Ol), and oleoyl-stearoyl-3-chloropropanediol (Ol-St); glycidyl esters standards, including glycidyl palmitate (Pa-GE), glycidyl linoleate (Li-GE), glycidyl oleate (Ol-GE), and glycidyl stearate (St-GE), were purchased from Toronto Research Chemicals (Toronto, Ontario, Canada). The purity of all the above standards was no less than 98.0%.

Stock solutions of 3-MCPDE (1000 mg/L) and internal standards (250 mg/L) were prepared using ethyl acetate and stored at −20 °C. The mixed stock solutions (Pa-Pa, Ol-Pa, Ol-Ol, Ol-St, Pa-GE, Ol-GE, St-GE, and Li-GE at 10 mg/L) were prepared by appropriate dilution of the corresponding stock solutions with IPA. The mixed internal standard solutions (St-GE-*d*_5_, Pa-Pa-*d*_5_, Ol-Ol-*d*_5_, and Ol-GE-*d*_5_ at 5 mg/L) were prepared by diluting the internal stock solutions with IPA. All solutions were stored at −20 °C.

### 2.2. Samples and Pre-Treatment

Palm oil, fresh chicken breast and potato were acquired from a local market. Potatoes were washed with water and cut into sticks of approximately 1 × 1 cm cross-section and 5–10 cm in length. The sticks were submerged in either 0 or 2% NaCl solution for 1 h. The sticks were then blanched in NaCl solutions with concentrations corresponding to the previous step for 90 s. The two samples were recorded as PS-0% NaCl and PS-2% NaCl. The fresh chicken breast was cut into 3 × 3 cm pieces and submerged in 0 or 2% NaCl solutions for 1 h. The two samples were recorded as CB-0% NaCl and CB-2% NaCl.

### 2.3. Deep-Fat Frying

Palm oil (3.5 L) was poured into a fryer. The temperature was raised to the corresponding temperatures in 10 min and maintained for 1.0 h before frying. The pre-treated samples (100 g) were fried for 2.0, 1.5, 1.0, and 1.0 min at the corresponding temperatures of 160, 180, 200, and 220 °C. Each hour was a cycle. Potato chips or chicken breasts were fried once in every cycle. In frying frequency parts, chicken breasts were fried once, three times, and five times, respectively. Eight cycles were performed per day. The oil was used continuously for 3 days without any replenishment. At the end of each 4 h period, 20 mL of oil was collected and stored in a freezer at −20 °C before analysis.

### 2.4. Fatty Acid Analysis

Free fatty acids were methylated and analyzed by gas chromatography based on the Chinese National Standard GB5009.168-2016 [19]. The frying oil samples were saponified and methylated in alkaline condition to form fatty acid methyl ester, which was analyzed by gas chromatographic–mass spectrometry (GC-MS) and quantitatively determined by external standard method.

### 2.5. Quantitation of TPC

TPC were determined using the Chinese National Standard GB5009.202-2016 [20]. The oil sample was separated into two parts: the non-polar and polar fractions after column chromatography (packed with Silica gel 60). The non-polar components were eluted with a mixture of petroleum and diethyl ether (87:13, *v*:*v*), evaporated to nearly dry, and finally vacuum dried to a constant weight; this weight indicated the content of non-polar components. The polar fraction was the remainder of the oil sample: the weight of the original sample minus the content of the non-polar fraction.

### 2.6. Color Analysis

The colors of the fresh oils and the oils after frying were determined using an UltraScan PRO system (Hunter Associates Laboratory, Inc., Reston, VA, USA). The differences in the colors of the samples were determined based on the different L, a, and b configurations in the color system. The color values were expressed as L (whiteness or brightness/darkness), a (redness/greenness), and b (yellowness/blueness).

The total color difference (Δ*E*) was used to evaluate the changes at different frying times. The values of Δ*E* were calculated using the following equation

(1) $$\Delta E=\sqrt{{\left(L_{0}-L_{f}\right)}^{2}+{\left(a_{0}-a_{f}\right)}^{2}+{\left(b_{0}-b_{f}\right)}^{2}}$$

where $L_{f}$, $a_{f}$, $b_{f}$, represents the color values of thermally treated oil, and $L_{0}$, $a_{0}$, $b_{0}$, represents the color values of initial oil.

### 2.7. Determination of 3-MCPDE and GE

A portion of the oil sample (0.1000 g) was accurately weighed into a sample bottle, and then 20 μL of internal standard and 980 μL of isopropanol were added. After the sample bottle was vortexed thoroughly, the sample was analyzed using HPLC-MS/MS.

Separation analysis was performed on a 1260 HPLC system (Agilent Technologies, Santa Clara, CA, USA) with an Eclipse Plus C18 (2.1 mm × 150 mm, 5 μm; Agilent Technologies). The column temperature was maintained at 40 °C. Mobile phase A consisted of methanol/water (92:8, *v*:*v*) with 0.05% formic acid and 1.0 mmol/L ammonium formate; the mobile phase B was composed of isopropanol/water (98:2, *v*:*v*) with 0.05% formic acid and 1.0 mmol/L ammonium formate. The gradient started at 100% A for the first 2 min, a linear ramp to 75% A at 8 min, followed by 30% at 15 min, and then 17% A for 20 min, followed by a linear decrease to 0% A at 25 min, and, finally, re-equilibration for 2 min before the next injection. The flow rate was 0.30 mL/min, and the injection volume was 5.0 μL.

A 6470 Triple Quadrupole Mass Spectrometer (Agilent Technologies) equipped with an electrospray ionization source (ESI) was used in positive mode. Analyst Lab Solutions LC/MS version B.09.00 software was used for the master control of LC and MS. The atomizing gas was nitrogen (N_2_ > 99.999%) at 300 °C. The ion source parameters were as follows: capillary voltage, 3.5 kV; gas temperature, 350 °C; drying gas flow, 5.0 L/min; nebulizer pressure, 0.31 MPa; sheath gas temperature, 350 °C; sheath gas flow (N_2_ > 99.999%), 11.0 L/min.

The retention times of the target compounds were determined by analyzing a mixed standard under the same conditions described above using the dynamic multiple reaction monitoring (dMRM) mode. The precursor–product ion transitions and optimized mass spectrometric parameters, such as the collision energy (CE), are listed in Table 1.

### 2.8. Kinetics and Statistical Analysis

In previous studies, the MCPDE or GE increased linearly with time (zero-order kinetics) according to the following equation [21].

(2) $$C_{t}=C_{0}+kt$$

where *C*_0_ is the initial content of GE or MCPDE (mg/kg), *t* is the heat treatment time (h), *C_t_* is the content at time *t* (mg/kg), and *k* is the rate constant (h^−1^).

The relative root mean square error (RRMSE) represented the sample standard deviation of the differences between the predicted and observed values [22]. The smaller the RRMSE value in comparison to the actual value, the better the goodness of fit. The RRMSE was calculated using the following equation

(3) $$\mathrm{RRMSE}\left(\%\right)=\frac{1}{{\bar{y}}_{A}}\sqrt{\frac{\sum_{i=1}^{n}{\left(y_{A}-y_{M}\right)}^{2}}{n}}\times 100$$

The temperature dependence of simple chemical reactions is empirically described by Arrhenius’ law, which is expressed as Equation (4)

(4) $$k=Ae^{-\frac{E_{a}}{RT}}$$

where *k* is the reaction rate constant, *A* is a pre-exponential factor, *Ea* is the activation energy, *T* is the absolute temperature, and *R* is the molar gas constant (8.314 kJ/mol·K).

According to Arrhenius’ equation (Equation (4)), the activation energy (*Ea*) and Arrhenius prefactor (*A*) are expressed as a linear equation, and, thus, the logarithm of the rate constant (ln*k*) against the reciprocal of the absolute temperature (1/T) is shown in Equation (5).

(5) $$\ln k=\ln A-\frac{E_{a}}{RT}$$

Data are presented as mean ± standard deviation (SD) from duplicate experiments using Excel 2007 (Microsoft Corp, Seattle, WA, USA). The Pearson range tests were used to determine the significant correlation coefficients (*p* ≤ 0.05) between 3-MCPDE, GE, and some of the examined parameters (TPC and color analysis). The 3-MCPDE and GE content in all figures were analyzed by one-way analysis of variance and Duncan’s multiple tests to identify significant differences (*p* ≤ 0.05) in the comparison of means. All analyses were performed using SPSS 20.0 (SPSS Inc., Chicago, IL, USA). All plots were determined using Origin 9.0.0 (Northampton, MA, USA).

## 3. Results

### 3.1. HPLC-MS/MS Analysis

The advantage of the direct analysis method is simplicity, and provides information about the fatty acid composition of 3-MCPDE and GE; however, a large number of standards are used during the analysis [23]. To achieve a balance between cost minimization and accurate quantitation, the fatty acid profiles of the palm oil were analyzed and are shown in Table S1. The predominant fatty acids were palmitic and oleic acids, which accounted for more than 80% of the fatty acids, followed by linoleic and stearic acids. Other fatty acids occurred to a lesser extent. Palmitic acid was the primary fatty acid in palm oil, whereas it was less abundant in other oils. The fatty acid composition of palm oil was similar to that of crude palm oil and preneuralized crude palm oil analyzed by Chaijan [24]. Therefore, the content of 3-MCPDE, consisting of palmitic and oleic acids, was analyzed in the oil samples.

For HPLC-MS/MS analysis, the dMRM model was used for scanning with one quantitation and two identification ions to avoid false-positive responses. The 3-MCPDE and GE were identified by their retention times and monitored characteristic ions (Table 1).

Standard solutions with different concentrations (0.05, 0.10, 0.20, 0.50, and 1.00 mg/L for each GE; 0.010, 0.020, 0.050, 0.10, 0.20, 0.50, and 1.00 mg/L for every 3-MCPDE) and internal solution with 0.100 mg/L for both deuteration GE and 3-MCPDE were analyzed as the samples. Thereafter, the calibration curves were obtained using a least-squares linear regression analysis of the ratio of target standards to internal peak area versus the ratio of the corresponding concentrations. The calibration curves of the four target 3-MCPDE were linear in the range of 0.010–1.00 mg/L, and those of the four target GEs were linear in the range of 0.050–1.00 mg/L, with a correlation coefficient no less than 0.99, as shown in Table S2. The limits of detections (LODs) and limits of quantifications (LOQs) of the method were calculated as the analyte concentrations with signal-to-noise (S/N) ratios of 3 and 10, respectively. The S/N ratios were estimated based on the peak heights of the identified ions. The LODs for 3-MCPDE and GE were in the ranges of 0.0002–0.0003 mg/kg and 0.0004–0.0100 mg/kg, respectively. The LOQs for 3-MCPDE and GE were 0.0004–0.010 mg/kg and 0.0050–0.0500 mg/kg, respectively.

To establish the suitability of the above method for the determination of 3-MCPDE and GE in edible oils, the accuracy and repeatability of the analysis method were determined. The chicken breast fried for 12 h in oil was chosen as the spiked sample, to which 1.00 to 3.00 mg/kg standard solutions were added. The extracted ion chromatogram was shown in Figure S1. Each spiked sample was subjected to six trials. The average recoveries of 3-MCPDE and GE ranged from 81.3 to 106.4% (0.90−8.1% RSD). The results of the recovery experiments (Table S3) showed that the overall average recoveries and repeatabilities (RSD) were 84.9 to 103% and 0.90 to 7.3% for 3-MCPDE, and 81.3 to 106.4% and 3.2 to 8.1% for GE, respectively.

### 3.2. Influence of Foods Types

**Figure 1:**
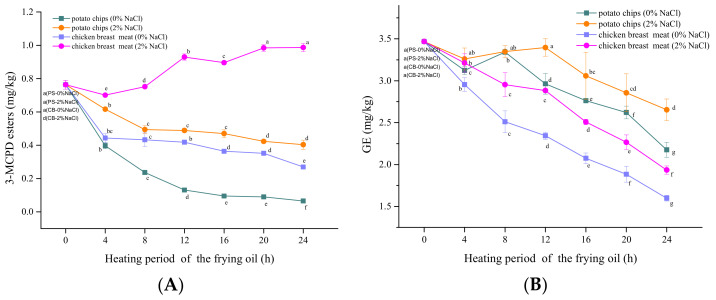
The content of (**A**) MCPDE and (**B**) GE fried oils of different heating periods after frying potato chips or chicken breast. Within each line, points with different letters are significantly (*p* < 0.05) different from each other.

Previous studies have indicated that the concentrations of 3-MCPDE and GE vary in different frying foods [25]. In the present study, potatoes and chicken breasts were used as representatives of high-carbohydrate and high-protein foods, respectively. As shown in Figure 1, the effect of food type on the changes of 3-MCPDE and GE content were significant. The GE content in the oil after frying chicken breasts decreased more than that after frying potato chips. The decline rate of GE was lower in the presence of NaCl, and the GE content in the frying oil decreased faster after frying CB-2% NaCl than that after frying PS-2% NaCl. The content of 3-MCPDE in the oil decreased the least after frying PS-2% NaCl, followed by that after frying CB-0% NaCl and PS-0% NaCl, which decreased the most. Interestingly, the 3-MCPDE content increased in the frying oil used for CB-2% NaCl. In general, MCPDE formed more easily in the oil used for frying chicken than that used for frying potatoes, while GE did the opposite. However, this might be because the chicken has more 3-MCPDE precursors. For example, chickens contain more lipids, but potatoes have almost no lipids. Furthermore, researchers have found that the total fatty acids are approximately 2.5 g/100 g in skinless chicken breast [26]. Fatty acids can form DAG, which has been identified as a precursor of MCPDE [27]. In contrast, potatoes contain several antioxidants, such as vitamins (B1, B6, B9, and C), carotenoids, and phenylpropanoids [28], which may inhibit the formation of MCPDE and GE. Although these compounds have not been confirmed to inhibit MCPDE and GE formation, other antioxidants have proven their inhibiting capacity, including butylated hydroxytoluene (BHT), butylated hydroxyanisole (BHA), tert-butyl hydroquinone (TBHQ), propyl gallate (PG), L-ascorbyl palmitate (AP), and α-tocopherol (VE) [29].

The content of 3-MCPDE was significantly higher in the CB-2% NaCl frying oil than that of CB-0% NaCl. This may be because the chloride anion can react with acylglycerol to form 3-MCPDE. This phenomenon has also been observed in other studies. For example, when the proportion of NaCl solution was increased from 0 to 60 g/100 g, the amount of 3-MCPDE sharply increased [7]. However, the formation rate of 3-MCPDE was lower than that of frying potatoes, as shown in Figure 1. Therefore, there was an overall decreasing trend for 3-MCPDE in the potato frying model.

Although the contents of GE were decreased both in the oils of frying potato chips and chicken breasts, the content of GE during frying of the samples with 2% NaCl was lower than that of the samples without NaCl, despite the formation of GE without chloride, unlike NaCl in 3-MCPDE. This phenomenon may be caused by chlorine-containing compounds, which promote the formation of GE. Although 3-MCPDE and GE can be transformed to each other, the transformation rate from MCPDE to GE is much higher [10].

### 3.3. Relationships between the Contents of 3-MCPDE/GE and TPC/Color

The analysis of 3-MCPDE and GE was complicated and time-consuming, and the assessment of TPC or the total color difference (Δ*E*) was relatively easy. TPC and color can be used as standards to evaluate oil quality [30,31]. In China, GB 7102.2-2003 stipulates that the TPC level in frying oil must be below 27%. The color of the frying oil darkens with increasing frying time [31]. Previous studies have used Pearson correlation analysis to assess the relationship between TPC levels and 3-MCPDE concentrations in fish processing [12]. However, the research was not comprehensive regarding the relationships between 3-MCPDE or GE and TPC or color values with different types of food and concentrations of NaCl during frying. In this case, the TPC content and the total color difference (Δ*E*) were analyzed, and the relationships between TPC or ΔE and 3-MCPDE or GE were determined by Pearson correlation analysis.

The graph (Figure S2) shows that Δ*E* increases as the frying time increases. There was a significant correlation (*p* < 0.05) between Δ*E* and frying time with the oil becoming darker as frying time increased. Other researchers have also noted the changes in the color of the oil during frying. For instance, Abdulkarim considered that the color value of red and yellow in edible oil increased after 5 days of frying [32]. As shown in Table 2, high correlation coefficients (*r* = 0.866–0.941) were observed during the frying process between the 3-MCPDE content and color value. The GE content in the oil used for frying chicken breast also correlated well with the color value (*r* = −0.895 to −0.943) of the frying oil. However, low correlation coefficients (*r* = −0.542 to −0.765) were observed between the GE content in the oil used for frying potato chips and the color value of the frying oil.

The determination of polar compound content in frying oils provides the most reliable index of the extent of oxidative degradation. The TPC content increased almost linearly with frying time (Figure S3). There was a significant correlation (*p* < 0.05) between TPC and frying time. During frying, new polar compounds are formed; thus, the proportions of DAG, MAG, and FFA increased. Significant correlation was also observed between the 3-MCPDE contents and TPC values (*r* = −0.892 to −0.961) and between GE contents and TPC values (*r* = −0.912 to −0.941) in most situations. However, the correlation between the 3-MCPDE content in the frying oil used for CB-2% NaCl and the TPC values was not statistically significant. Similarly, there was a non-significant correlation between the GE content in the frying oil of PC-2% NaCl and the TPC values.

Based on these results, it is suggested that both the TPC and the color values of the frying oils could be regarded as concentration prediction tools for 3-MCPDE and GE to a large extent, although not all of them were significantly correlated statistically. Furthermore, Merkle proposed that TPC and color values could be used as screening methods for estimating the amount of 3-MCPDE and GE in fish products [12]. Thus, color and TPC values could be used to estimate the contents of 3-MCPDE and GE, especially 3-MCPDE.

### 3.4. Influence of the Frying Frequency

According to the above experimental results, the GE content of palm oil declined during the frying process, while that of 3-MCPDE was only increased in the frying oil of CB-2% NaCl. Therefore, to study the formation rules of 3-MCPDE, CB-2% NaCl fried in palm oil was used as the target sample. Differences in frying frequency may be related to the degree of TAG hydrolysis during frying. However, no studies have discussed the relationships between frying frequency and the content of 3-MCPDE and GE. Therefore, the relationship between the frying frequency and the contents of 3-MCPDE and GE is further discussed.

**Figure 2:**
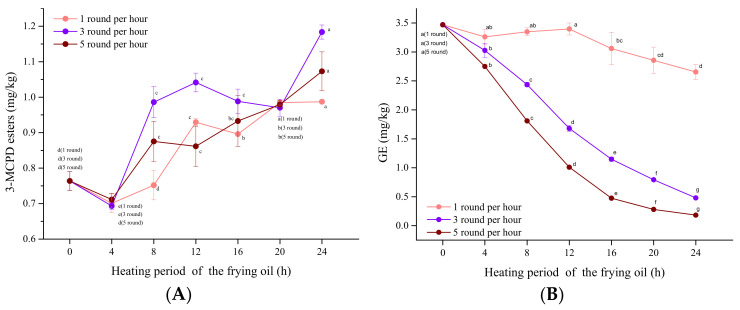
The content of (**A**) MCPDE and (**B**) GE fried oils of different heating periods after frying chicken breast 1, 3, or 5 times per hour. Within each line, points with different letters are significantly (*p* < 0.05) different from each other.

In the present study, three frying frequencies, one, three, and five times per hour, were considered in the experiment. The results are shown in Figure 2; the concentrations of 3-MCPDE in the three frying systems increased from 29.2 to 55.0% over the entire frying duration, although its content decreased for 0–4 h, regardless of frying frequency per hour. Unlike the linear increase in 3-MCPDE content with frying frequencies of one and five after 4 h, the content increase in 3-MCPDE after frying three times increased sharply from 4 to 12 h, then declined slightly after 12 h, and finally increased sharply at 20–24 h. It is worth noting that the percentage increased the highest (55.0%) when frying three times per hour. When the frying frequency increased from once to three times, more water molecules in the chicken breast may have reacted with TAG, causing an increase in TAG hydrolyzation. As mentioned above, TAG can be hydrolyzed to DAG and MAG, which are precursor substances of 3-MCPDE during frying. When the frying frequency was increased to five times, the water accelerated the degradation of 3-MCPDE. This phenomenon was also demonstrated by Zhou [33]. They found that 3-MCPDE increased significantly (approximately 3000 mg/kg) as the water content increased from 1 to 10% under palm oil frying. However, as the heating time increased, the 3-MCPDE content declined after 2 h.

As the frying frequency increased, the GE content declined steeply when frying three and five times (86.2–94.8%). A similar study found that GE was reduced by more than three quarters and one half in palm oil and rapeseed oil, respectively, during intermittent frying for 40 h [34]. Researchers have pointed out that GE is unstable and degrades more rapidly during high temperature exposure in 2 h [35], which explains the continuing decline of the GE content during the frying process.

### 3.5. Influence of Frying Temperature

**Figure 3:**
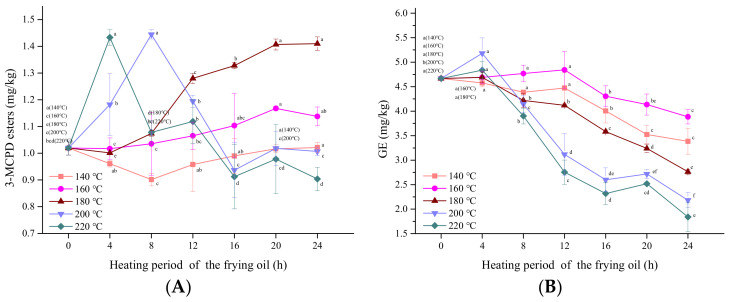
The content of (**A**) MCPDE and (**B**) GE fried oils of different heating periods after frying chicken breast under 140 °C, 160 °C, 180 °C, 200 °C, and 220 °C, respectively. Within each line, points with different letters are significantly (*p* < 0.05) different from each other.

According to the broken line graph shown in Figure 3, as the temperature increased from 140 to 180 °C, the content of 3-MCPDE increased in all frying for 24 h. At 200 and 220 °C, the contents of 3-MCPDE increased and then decreased, and the two maximum concentrations occurred at 4 and 8 h, respectively. Thus, high temperatures promoted 3-MCPDE formation to a certain extent. Similarly, when the temperature increased from 220 to 260 °C, the content of 3-MCPDE increased within 1.5 h during peanut oil deodorization [27]. During thermal processing, DAG, MAG, and other polar products (e.g., free fatty acids) from the hydrolyzed TAG can form acyloxonium ions, which react with chloride ions to form 3-MCPDE. Thus, higher temperatures could accelerate the hydrolysis of TAG, resulting in a high 3-MCPDE content. However, the high temperature affects not only the formation of 3-MCPDE but also its degradation. For instance, when pure 3-MCPDE was heated at temperatures from 180–260 °C, the degradation percentages ranged from 30 to 70% over 24 h [36].

The GE content declined at frying temperatures from 140 to 220 °C. The higher GE content was at relatively low frying temperatures (140–180 °C) than at higher frying temperatures (200–220 °C), as shown in Figure 3. This indicates that GE degraded more rapidly at higher frying temperatures. Recently, a similar study reported that GE levels decreased after 2 h at 200 °C in a DAG model in an ampoule bottle [35]. A review article [37] also pointed out that the degradation rate of GE was higher than its formation rate at higher temperatures.

### 3.6. Kinetics Analysis

To further elucidate the formation of MCPDE during frying, a kinetics analysis of 3-MCPDE was performed during heating at 140, 160, and 180 °C for 24 h, 200 °C for 8 h, and 220 °C for 4 h, in which 3-MCPDE was increased (Figure S4). Meanwhile, a kinetics analysis of GE was conducted during heating from 140 °C to 220 °C for 24 h. GE contents decreased as the frying temperature increased; therefore, the degradation kinetics of GE during frying were further established.

Researchers believe that glycerin ester is a precursor of GE and 3-MCPDE in vegetable oil. Previous studies revealed that the formation rate was affected by various types of oil. However, there are few articles illustrating the kinetics [38]. In the present study, each of the four 3-MCPDE and GE models was established by plotting (C-C_0_) against time. The results are shown in Table 3 where the linear curves fitted the data, indicating zero-order reaction kinetics for 3-MCPDE formation and GE degradation. From Table 3, the formation rate constants (k) of Ol-Pa, Ol-Ol, and Ol-St increased from 4.40 × 10^−3^ to 56.88 × 10^−3^/h, 1.98 × 10^−3^ to 22.83 × 10^−3^/h, and 2.03 × 10^−3^ to 23.13 × 10^−3^/h, respectively, with an increase in temperature (160–220 °C). The formation rate constant of Pa-Pa increased from 160 to 200 °C, except at 220 °C. This is because the formation and degradation of 3-MCPDE occurred simultaneously, and the degradation rate was higher than the formation rate at 220 °C compared with 200 °C. The degradation rate constant of the four types of GE increased with increasing temperature in the range of 140–220 °C, as shown in Table 3. A similar study also found that the degradation rate constant of GE increased from 1.21 to 2.95 h^−1^ with an increase in temperature from 120 to 240 °C, which is much higher than the formation rate constant [35].

To illustrate which glycerin esters were dominant, a further study was conducted comparing their corresponding Ea values. The Ea values for the different glycerin esters were calculated according to the Arrhenius equation (Equation (3)). Ea values and corresponding exponential factors of the four 3-MCPDE in order from high to low were 66.00 kJ/mol (exponential factor: 8.39 × 10^5^ h^−1^, the same as following), 65.09 kJ/mol (5.86 × 10^5^ h^−1^), 53.58 kJ/mol (7.46 × 10^4^ h^−1^), and 41.05 kJ/mol (6.89 × 10^5^ h^−1^) for Ol-St, Ol-Ol, Ol-Pa, and Pa-Pa, respectively. This implied that due to the lower Ea, palm glyceride easily formed 3-MCPDE compared with other types of glycerides, such as Ol and St. This could explain why palm oil was more inclined to form 3-MCPDE than other types of oil because a large proportion of palm glyceride exists in palm oil. Some researchers have also shown that the content of 3-MCPDE is higher in palm oil than in other types of oil during deodorization [39,40]. Based on the molecular reaction mechanism, the energy barriers for the formation of Pa-Pa, Ol-Ol, and Ln-Ln were 81.012, 83.896, and 79.109 kJ/mol, respectively, under water conditions. Pa-Pa is formed more easily than Ol-Ol because it crosses the energy barrier more easily at high temperatures [11].

The Ea of Li-GE, Pa-GE, St-GE, and Ol-GE was 18.49, 24.88, 20.89, and 32.06 kJ/mol, respectively. They are all lower than the formation Ea of 34.58 kJ/mol during the formation process [35]. Based on these results, it can be speculated that the degradation of GE is dominant compared with its formation, especially in Li-GE because it has the lowest Ea value. In addition, according to the above Ea values, there are different degradation rates in different types of GE. Unlike the formation of GE, the degradation reaction mechanism is not mediated by free radicals. At high temperatures, GE is degraded into MAG, fatty acid, and glycerol via a ring-opening reaction and continues to be quickly decomposed into hydrocarbons, aldehydes, and CO_2_ in addition to polar compounds [41].

## 4. Conclusions

The present study established a direct analysis method with good accuracy and precision using HPLC-MS/MS with dMRM for 3-MCPDE and GE. The formation of 3-MCPDE was affected by the NaCl content, temperature, and time in palm oil during frying. 3-MCPDE is formed more easily in chicken breasts than in potato chips. The GE content in frying oils decreased with increasing frying time during chicken breast and potato chip frying. The frying frequency had an important effect on the formation of 3-MCPDE and the decrease in GE in frying oils. The formation of 3-MCPDE and degradation of GE followed zero-order reactions. Pa-Pa is more likely to form than the other three 3-MCPDE, and Li-GE is easily degraded because of its low Ea values. The content of 3-MCPDE in palm oil is higher than that in other edible oils because of the high content of palmitic glycerides. This study provides a novel insight into the formation of 3-MCPDE and degradation of GE, which offers a theoretical basis for the efficient inhibition of 3-MCPDE and GE in foods.

## Figures and Tables

**Table 1:** MS parameters for GE and 3-MCPDE.

Compounds	Abbreviation	RT (min)	Precursor Ion [M + NH_4_] (*m*/*z*)	Production (*m*/*z*)	Collision Energy (eV)	Internal Standard
GE						
Glycidyl linoleate	Li-GE	3.88	354.3	81.1 *	32	Ol-GE-*d*_5_
				95.1	28	
Glycidyl palmitate	Pa-GE	4.77	330.3	57.2 *	32	Ol-GE-*d*_5_
				71.1	24	
Glycidyl oleate	Ol-GE	5.31	356.3	57.1 *	32	Ol-GE-*d*_5_
				69.1	36	
Glycidyl stearate	St-GE	7.65	358.3	57.2 *	36	St-GE-*d*_5_
				71.1	32	
3-MCPDE						
1,2-Bis-palmitoyl-3-chloropropanediol	Pa-Pa	18.87	604.5	331.1 *	14	Pa-Pa-*d*_5_
				239.2	20	
Oleoyl-palmitoyl-3-chloropropanediol	Ol-Pa	19.05	630.5	331.3 *	20	Pa-Pa-*d*_5_
				357.3	20	
1,2-Bis-oleoyl-3-chloropropanediol	Ol-Ol	19.21	656.5	357.3 *	22	Ol-Ol-*d*_5_
				265.2	22	
Oleoyl-stearoyl-3-chloropropanediol	Ol-St	19.23	658.5	359.3 *	20	Ol-Ol-*d*_5_
				357.3	24	
Internal standard						
Glycidyl oleate-*d*_5_	Ol-GE-*d*_5_	5.23	361.0	69.3 *	40	
				83.2	28	
Glycidyl stearate-*d*_5_	St-GE-*d*_5_	7.56	363	57.2 *	36	
				85.1	28	
1,2-Bis-palmitoyl-3-chloropropanediol-*d*_5_	Pa-Pa-*d*_5_	18.82	609.3	239.2	24	
				336.3 *	12	
1,2-Bis-oleoyl-3-chloropropanediol-*d*_5_	Ol-Ol-*d*_5_	19.18	661.53	362.3 *	28	
				265.1	20	

* quantitative ion.

**Table 2:** Correlation analysis between 3-MCPDE or GE and TPC or Δ*E* value.

	Correlation (*r*) of MCPD Esters and GE in the Frying Oil (µg/kg) with ΔE Value		Correlation (*r*) of MCPD Esters and GE in the Frying Oil (µg/kg) with the TPC Value [%]		Correlation of ΔE in the Frying Oil with the Different Heating Hour	Correlation of TPC in the Frying Oil with the Different Heating Hour
	3-MCPDE	GE	3-MCPDE	GE		
Potato chips (0% NaCl)	−0.992 **	−0.765	−0.892 *	−0.916 *	0.937 **	0.973 **
Potato chips (2% NaCl)	−0.954 **	−0.542	−0.935 *	−0.811	0.866 *	0.991 *
Chicken breast meat (0% NaCl)	−0.781	−0.943 **	−0.961 *	−0.912 *	0.909 *	0.956 *
Chicken breast meat (2% NaCl)	0.949 *	−0.895 *	0.857	−0.990 **	0.941 **	0.979 *

* significant correlation coefficients (*p* ≤ 0.05); ** extremely significant coefficients (*p* ≤ 0.01).

**Table 3:** Kinetic parameters of 3-MCPDE formation and GE degradation at various temperatures (140−220 °C) in palm oil during frying chicken breast.

		T(℃)					Arrhenius Equation		
		140	160	180	200	220	A (h^−1^)	Ea (kJ/mol)	R^2^
Pa-Pa	K (×10^−3^, h^−1^)	-	2.66	4.06	14.61	10.41	6.89 × 10^5^	41.05	0.81
	R^2^	-	0.914	0.953	0.861	0.863			
	RRMSE%	-	22.82	14.57	29.84	15.42			
Ol-Pa	K (×10^−3^, h^−1^)	4.22	4.40	9.36	46.14	56.88	7.46 × 10^4^	53.58	0.89
	R^2^	0.937	0.85	0.958	0.794	0.863			
	RRMSE%	17.49	20.24	7.51	12.28	23.16			
Ol-Ol	K (×10^−3^, h^−1^)	-	1.98	2.84	10.93	22.83	5.86 × 10^5^	65.09	0.95
	R^2^	-	0.884	0.676	0.865	0.878			
	RRMSE%	-	20.40	30.14	28.83	14.10			
Ol-St	K (×10^−3^, h^−1^)	-	2.03	3.18	14.03	23.13	8.39 × 10^5^	66.00	0.93
	R^2^	-	0.835	0.832	0.843	0.884			
	RRMSE%	-	26.83	22.27	29.66	13.23			
Li-GE	K (×10^−3^, h^−1^)	19.11	26.76	30.92	52.38	49.29	7.06	18.49	0.93
	R^2^	0.99	0.966	0.988	0.823	0.947			
	RRMSE%	6.86	25.95	4.46	27.09	12.10			
Pa-GE	K (×10^−3^, h^−1^)	16.85	16.48	23.86	59.04	50.57	38.91	24.88	0.81
	R^2^	0.925	0.893	0.828	0.951	0.962			
	RRMSE%	5.52	22.06	16.15	26.12	16.49			
St-GE	K (×10^−3^, h^−1^)	7.42	6.00	9.17	37.23	34.01	2.01	20.89	0.96
	R^2^	0.931	0.958	0.805	0.958	0.949			
	RRMSE%	14.53	17.41	17.97	16.29	21.04			
Ol-GE	K (×10^−3^, h^−1^)	2.44	3.52	5.89	6.28	7.72	145.22	32.06	0.72
	R^2^	0.806	0.945	0.945	0.925	0.951			
	RRMSE%	28.58	24.28	9.06	21.06	21.80			

## Data Availability

Not applicable.

## Supplementary Materials

The following are available online at https://www.mdpi.com/article/10.3390/foods10102266/s1, Figure S1: Extracted ion chromatogram of: (**a**) Li-GE, (**b**) Pa-GE, (**c**) Ol-GE, (**d**) St-GE, (**e**) Pa-Pa, (**f**) Ol-Pa, (**g**) Ol-St, (**h**) Ol-Ol in the fried palm olein sample after frying for 4 h at 200 °C, Figure S2: Δ*E* value in fried oils of different heating period after frying potato chips and chicken breasts, Figure S3: TPC amount in fried oils of different heating period after frying potato chips and chicken breasts, Figure S4: Contents of 3-MCPDE in fried oils after frying chicken breasts at 220 °C for 0–4 h and at 200 °C for 0–8 h, Table S1: Fatty acid profile (g/100 g lipid) of palm oil, Table S2: Calibration curves, correlation coefficients (*r*), LODs and LOQs for GEs and 3-MCPDE, Table S3: Recovery and repeatability results of GEs and 3-MCPDE analysis.
