# Supplementary material for: Effects of Food Types, Frying Frequency, and Frying Temperature on 3-Monochloropropane-1,2-diol Esters and Glycidyl Esters Content in Palm Oil during Frying
Source: Foods. 2021 Sep 24;10(10):2266. doi: 10.3390/foods10102266 (PMC8534808; doi:10.3390/foods10102266)
Supplement: Supplementary file 1 [file foods-10-02266-s001.zip › Supplementary data-20211019.pdf]

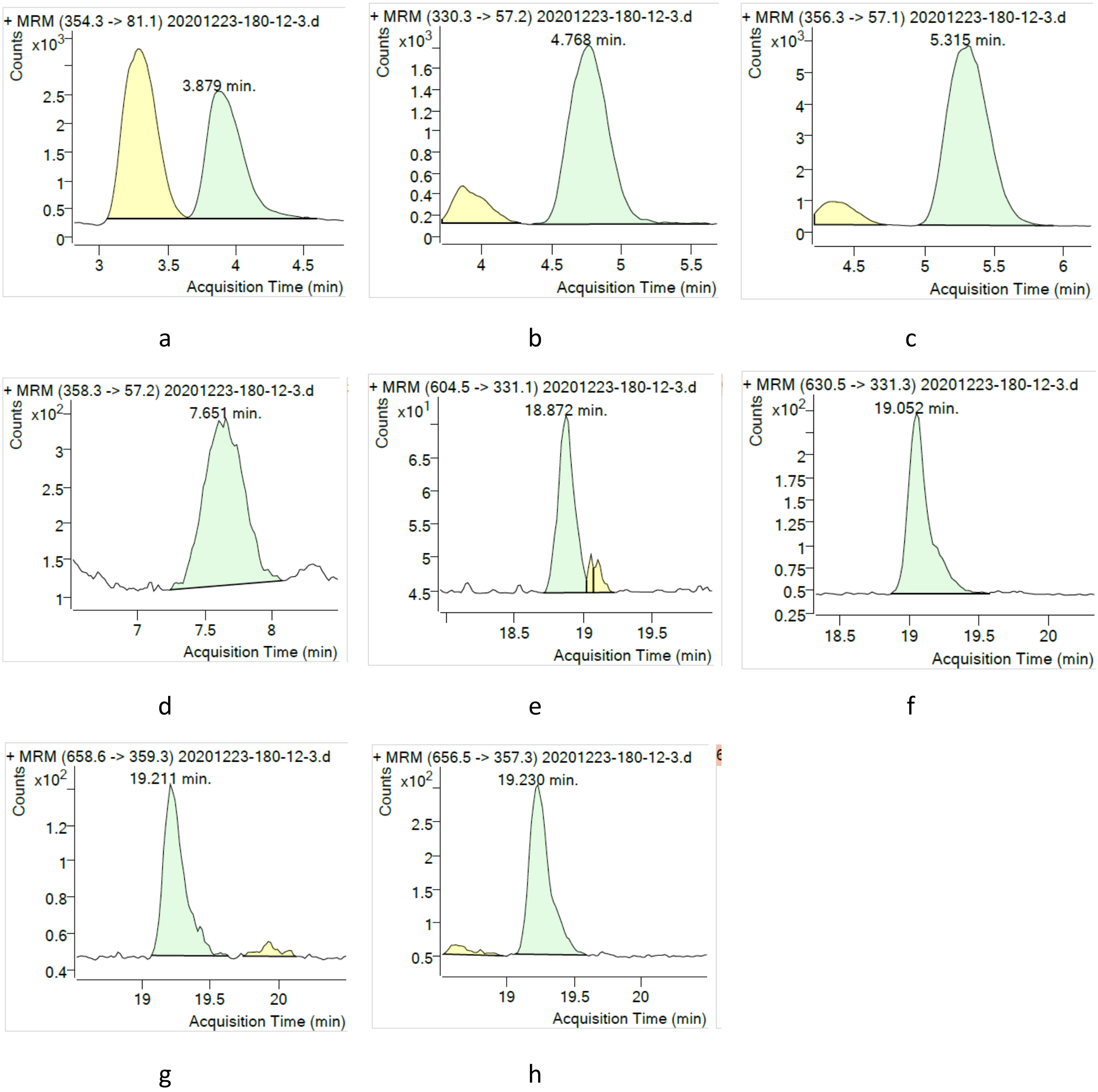

Figure S1. Extracted ion chromatogram of: (a) Li- GE, (b) Pa-GE, (c) Ol-GE, (d) St-GE, (e) Pa-Pa, (f) Ol-Pa, (g) Ol-St, (h) Ol-Ol in the fried palm olein sample after frying 12 h at 180℃

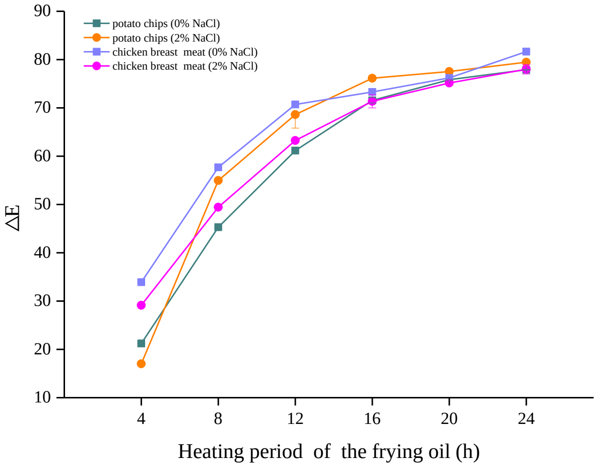

Figure S2. ΔE value in fried oils of different heating period after frying potato chips and chicken breast

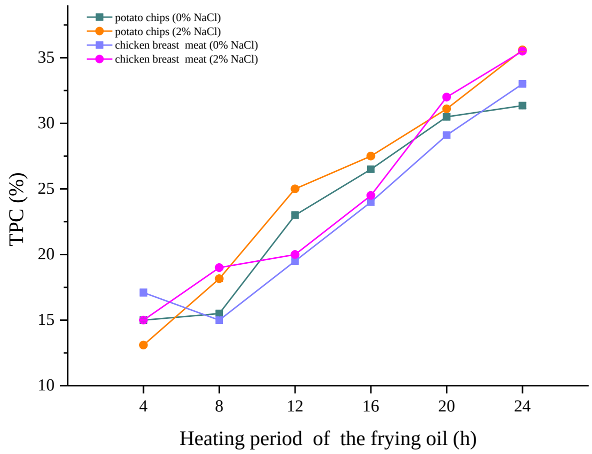

Figure S3. TPC amount in fried oils of different heating period after frying potato chips and chicken breast

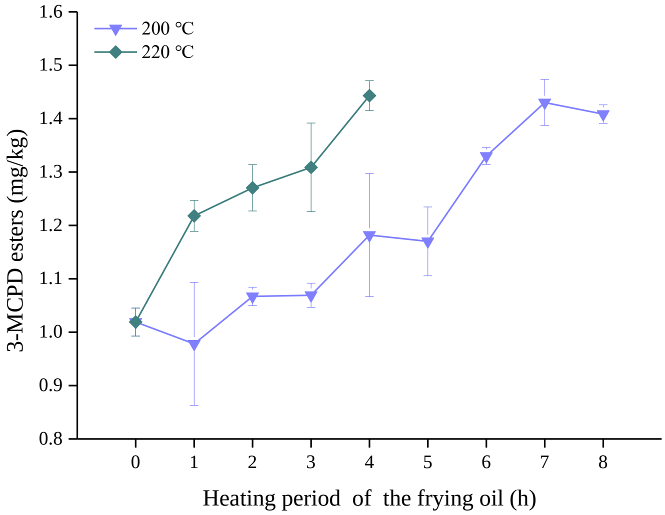

Figure S4. Contents of 3-MCPDE in fried oils after frying chicken breast at 220℃ for 0-4h and at 200℃ for 0-8 h

Table S1. Fatty acid profile (g/100 g lipid) of palm oil

Fatty acids	Contribution* (relative percentages)
Lauric acid (C12:0)	0.16±0.01
Tetradecanoic acid (C14:0)	0.68±0.01
Palmitic acid (C16:0)	29.10±0.10
Palmitoleic acid (C16:1)	0.15±0.01
Stearic acid (C18:0)	2.68±0.01
cis-9-Oleic acid (C18:1)	49.50±0.30
Linoleic acid (C18:2)	13.20±0.10
cis-11-Ecosenoic acid (C20:1)	0.18±0.01
Linolenic acid (C18:3)	0.20±0.01
Total	95.85

*All values are averages ± standard deviation of triplicate analysis

Table S2. Calibration curves, correlation coefficients (r), LODs and LOQs for GEs and 3-MCPDE

Compounds	Linear range (mg/L)	R^2	Calibration equation	LOQ (mg/kg)	LOD (mg/kg)
Li-GE	0.05-1	0.9998	y = 5.292x - 0.063	0.020	0.004
Pa-GE	0.05-1	0.9989	y = 1.771x + 0.070	0.005	0.0004
Ol-GE	0.05-1	0.9980	y = 10.865x + 1.270	0.020	0.004
St-GE	0.05-1	0.9984	y = 0.844x + 0.069	0.050	0.010
Pa-Pa	0.01-1	0.9992	y = 1.037x - 0.046	0.001	0.0002
Ol-Pa	0.01-1	0.9942	y = 2.067x - 0.373	0.0004	0.0002
Ol-Ol	0.01-1	0.9991	y = 0.867x - 0.045	0.0004	0.0002
Ol-St	0.01-1	0.9991	y = 0.314x - 0.011	0.001	0.0003

Table S3. Recovery and repeatability results of GEs and 3-MCPDE analysis

Compounds	Spiked level (mg/kg)	*Recoveries (%, n=6)	RSD (%)
Li-GE	1	96.5	5.7
	3	81.3	4.1
Pa-GE	1	93.1	6.0
	3	84.5	3.2
Ol-GE	1	101.4	5.0
	3	106.4	3.6
St-GE	1	95.0	8.1
	3	91.4	4.6
Pa-Pa	1	84.9	7.3
	3	89.7	5.1
Ol-Pa	1	103.0	6.7
	3	95.9	6.7
Ol-Ol	1	92.5	3.9
	3	94.7	2.0
Ol-St	1	89.0	2.7
	3	90.8	0.9

*The recoveries are shown as the mean from six trials
